# Postmorterm Computed Tomography and Autopsy to Confirm Sudden Death Due to Tracheal Compression by Mediastinal Fat Tissue in a Young Man With Obesity

**DOI:** 10.7759/cureus.33322

**Published:** 2023-01-03

**Authors:** Hideyuki Maeda, Keiko Matsuno, Yoshiteru Tamura, Shigeo Sogabe, Ken-ichi Yoshida

**Affiliations:** 1 Forensic Medicine, Tokyo Medical University, Tokyo, JPN; 2 Autopsy, Osaka Prefectural Office of Medical Examiner, Osaka, JPN; 3 Radiology, Osaka Prefectural Office of Medical Examiner, Osaka, JPN; 4 Forensic Medicine, Osaka Prefectural Office of Medical Examiner, Osaka, JPN

**Keywords:** tracheal obstruction, postmortem computed tomography, obesity medicine, mediastinal tumors, hypoxia

## Abstract

A man in his early twenties with obesity was found dead in his apartment. The deceased was found naked and surrounded by empty bottles of electrolytes. An autopsy performed approximately 6 days postmortem and gross inspection revealed the absence of injury and no apparent extrinsic cause of death. It was decided to dissect to investigate the cause of death.

The deceased had become morbidly obese (weight, 98 kg; height, 160 cm; body mass index, 38.3). Shortly before his death, he presented at a clinic complaining of gastric discomfort and heartburn, but other than hypertension (155/91 mmHg) no specific abnormality was found. He was normothermic (36.6℃), and his blood oxygen saturation was normal (97%).

Postmortem computed tomography of the thorax revealed a mediastinal mass obstructing the trachea, an upper-airway obstruction, and a narrowed thoracic cavity due to upward compression by an enlarged fatty liver. Autopsy confirmed that the tracheal mass was fatty tissue within the thymus and that upward pressure from an enlarged fatty liver had compressed the thoracic cavity. The deceased likely developed nocturnal chronic hypoxia because of compression by the mediastinal fat mass as well as intermittent hypoxia because of obstructive sleep apnea when lying supine. Chronic and intermittent hypoxia, diabetes, and obesity activate the sympathetic nervous system, increasing the risk of hypertension, heart failure, and arrhythmias. Histological findings showed pulmonary congestion and edema, reflecting heart failure as well as myocardial fragmentation and waving, showing hyper-contraction and hyper-relaxation, respectively. Hypertension, feeling overheated, and myocardial hyper-contraction can be explained as sympathetic nerve over-activation. Intra-cardiac coagulation and a renal cortical pallor suggested subacute death from cardiogenic shock due to heart failure.

Postmortem computed tomography before autopsy detected airway obstruction and revealed the cause and pathophysiology of unexpected death in a young man with morbid obesity. Therefore, this could be a potentially useful clinical practice for determining the cause of death postmortem.

## Introduction

Both tracheal compression and deformation due to mediastinal masses in children [1] and tracheal neoplasms in adults [2] have been observed by computed tomography (CT). The tracheal cross-sectional area was shown to correlate inversely with increasing subcutaneous fat thickness in a random population [3]. Expiratory collapse is associated with body mass index in patients who are morbidly obese with chronic obstructive pulmonary disease [4]. However, sustained tracheal obstruction by mediastinal fat masses in persons with no history of chronic disease has not been reported. Here, we describe the unexpected death of a young male with obesity and tracheal compression caused by a mediastinal fat mass in the supine position, upper airway obstruction due to obstructive sleep apnea (OSA), and a thoracic cavity narrowed by an enlarged fatty liver. These contributing factors were identified by CT imaging and autopsy, and they are known to induce hypoxia and sympathetic nerve activation [5].^ ^Diabetes and OSA are prevalent among persons with morbid obesity and precede hypertension, heart failure, arrhythmias, and sudden cardiac death via sympathetic activation [6,7].

Here, we discuss the determination of his cause of death and pathophysiology from postmortem CT (PMCT), autopsy, histological findings, and the known circumstances of death with respect to hypoxia, sympathetic activation, diabetes, and OSA.

## Case presentation

Patient information

A man in his early twenties with obesity was found dead in his apartment. The deceased was found naked, wearing two types of coolants, and surrounded by empty bottles of electrolytes. An air-conditioner was set at 20℃, indicating that he had felt overheated, although the day was not reported to be particularly hot by others in the vicinity. An autopsy performed approximately 6 days postmortem and gross inspection revealed the absence of injury and no apparent extrinsic cause of death. The deceased presented severe facial congestion, cyanosis, and hypostasis, suggesting death from circulatory and/or respiratory failure. Intra-cardiac coagulation and renal cortical pallor supported subacute death. This case report was approved by the institutional ethics committee and was prepared in accordance with “Protection Guideline of Personal Information on Research Publication in Legal Medicine”.

The deceased had become morbidly obese (weight, 98 kg; height, 160 cm; body mass index, 38.3) after entering college. He had played internet games for about 2 months after leaving a basketball club. Four days before his death, he presented at a clinic complaining of gastric discomfort and heartburn, but other than hypertension (155/91 mmHg) no specific abnormality was found. He was normothermic (36.6℃), and his blood oxygen saturation was normal (97%). He was prescribed famotidine (an H_2_ receptor antagonist and antacid) and itopride (an anti-dopaminergic agent) to improve his gastrointestinal systems.

PMCT findings

**Figure 1:**
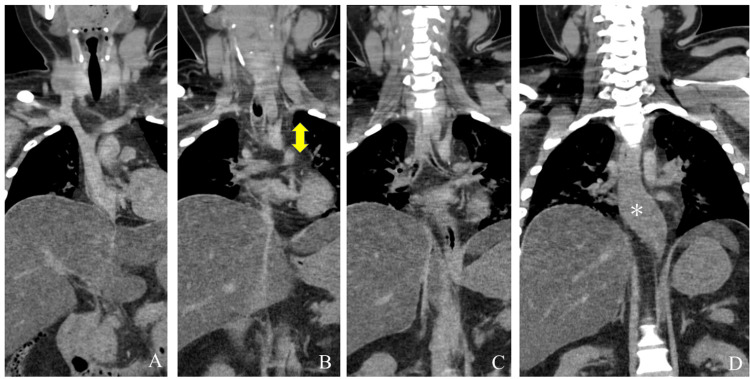
Coronal sections of thoracic cavity from anterior (A)-to-posterior (D) planes. The arrows in Figure 1B indicate that the lower trachea was compressed by a mediastinal mass. The asterisk in Figure 1D indicates that the lower esophagus was compressed and laterally widened with distal constriction.

Coronal CT sections of the thoracic cavity showed that the lower trachea was compressed by a soft mediastinal mass (Figure 1A and Figure 1B, bidirectional arrow). The bilateral bronchi were patent (Figure 1C). The lower esophagus was compressed and laterally widened with distal constriction (Figure 1D). The distal esophagus was constricted, possibly because of compression and a leftward shift caused by the enlarged liver.

**Figure 2:**
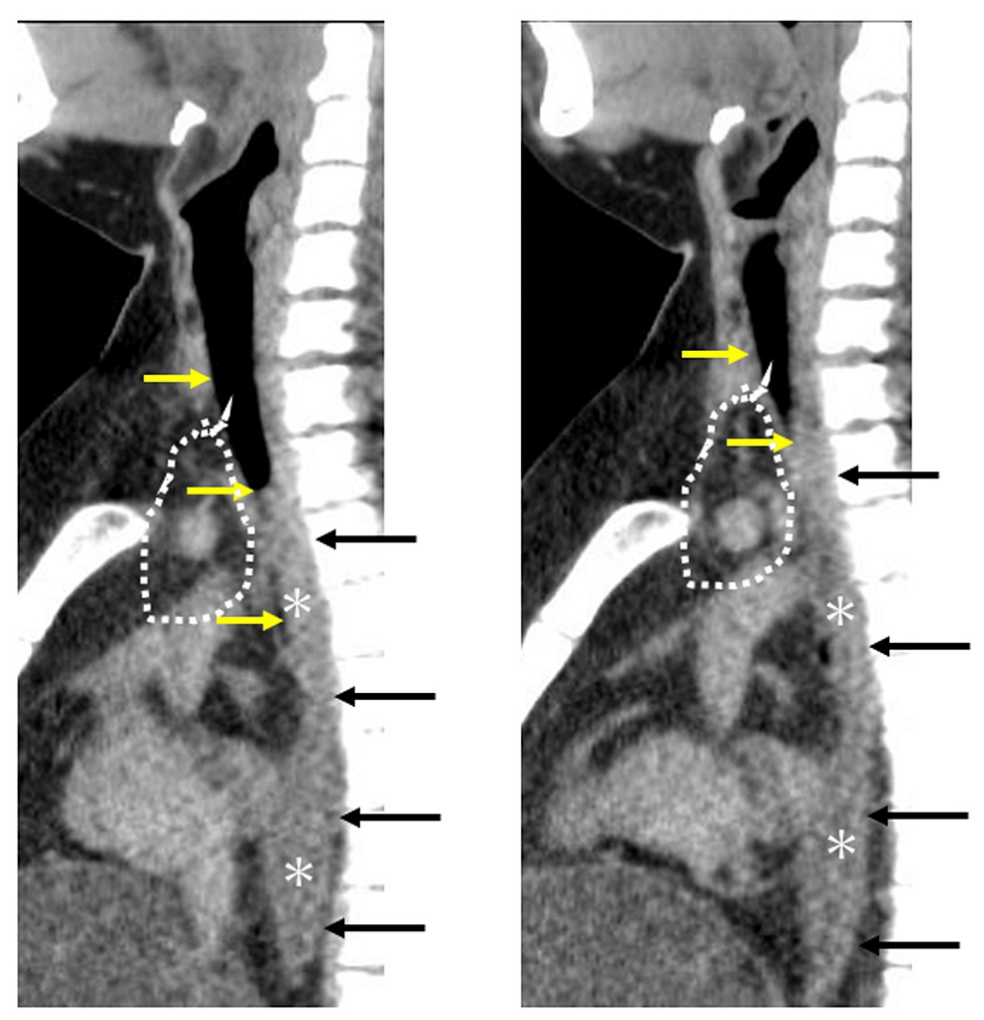
Sagittal section of the trachea and esophagus. The area encircled by a dotted line indicates that the lower trachea had been compressed between the thoracic vertebrae and upper mediastinal soft tissue. Asterisk indicates the compressed esophagus, and arrow indicates the direction of compression.

Sagittal CT sections showed that the lower trachea had been compressed between the thoracic vertebrae and upper mediastinal soft tissue (Figure 2; encircled by a dotted line). The esophagus was also compressed by the mediastinal soft tissue, trachea, and thoracic vertebrae, and the anteroposterior diameter was shorter than the transverse diameter. Posterior deviation and obstruction of the upper airway were also consistent with OSA.

**Figure 3:**
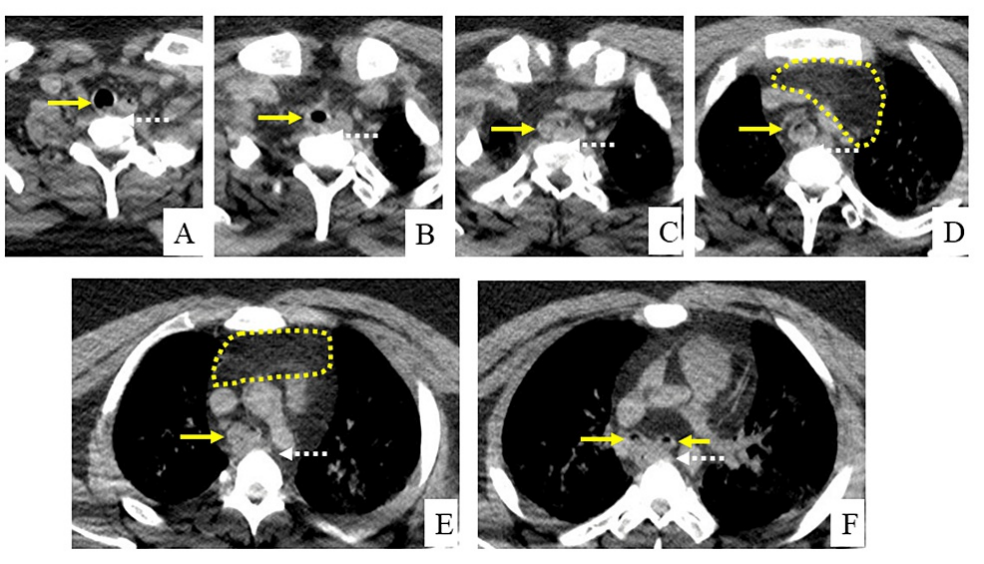
Axial sections of the thoracic cavity arranged from the upper (A) to lower (F) directions. The area encircled by a dotted line indicates that the lower trachea had been compressed between the thoracic vertebrae and upper mediastinal soft tissue. Arrows indicate the trachea and main bronchus.

Axial CT images confirmed that the lower trachea was compressed between the mediastinal soft tissue and thoracic vertebrae (Figure 3) and that the esophagus was compressed by the mediastinal soft tissue, trachea, and thoracic vertebrae.

Autopsy and histological findings

The trachea was soft and relatively small. It was flattened in the lower portion upon excision but recovered to a tubular morphology at the end of the autopsy. We assumed that compression by the soft tissue had induced the tracheal obstruction. No histological abnormalities were evident in the tracheal cartilage. The trachea contained a moderate amount of brownish murky material, similar to the stomach content (150 mL). The bilateral bronchi were patent.

The esophagus was moderately distended upon excision and it contained a small amount of murky stomach content. Neither macroscopic wall thickening, nor discoloration was found. CT findings of esophageal distention suggested achalasia, which is caused by degeneration of ganglion cells in the myenteric plexus of the esophageal body and lower esophageal sphincter and leads to an amotile esophageal body and non-relaxing lower esophageal sphincter [8]. However, histological findings did not support achalasia. We suspected that the esophageal content was swallowed air that had become trapped in the lower esophagus with the compressed upper esophagus acting as a check valve.

**Figure 4:**
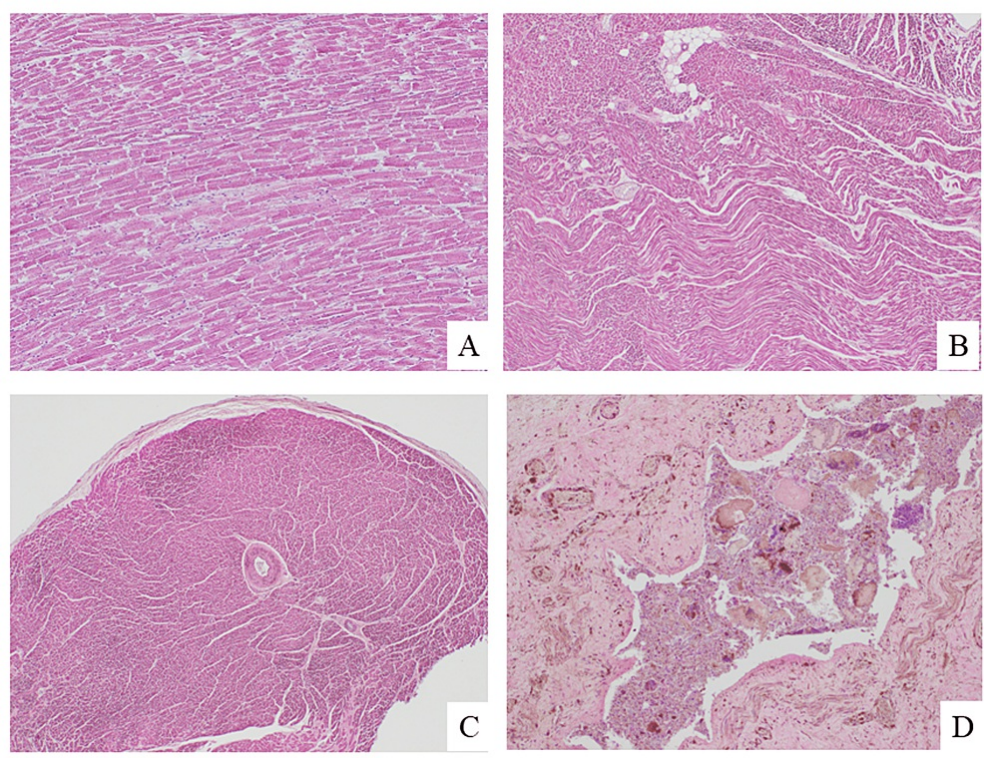
Histological sections of the heart (A, B, C) and the lung (D). Myocardial fragmentation and waving on the heart sections reflected hypercontraction and hyperrelaxation, respectively (A and B). The wall of the small intra-myocardial artery was thickened (C). The pulmonary section showed aspirated materials with bacterial packets, without an inflammatory reaction (D).

The heart weighed 390 g and showed a normal wall width and slight chamber dilation. Cardiac hypertrophy, coronary sclerosis, and myocardial fibrosis were not evident. Myocardial fragmentation and waving on the heart sections reflected hypercontraction and hyperrelaxation, respectively (Figure 4A and Figure 4B). These findings are prevalent in sudden cardiac death [9]. The wall of the small intra-myocardial artery was thickened (Figure 4C), consistent with diabetic micro-circulatory dysfunction [10]. Macroscopic and microscopic findings of the lungs (left and right, 455 and 475 g, respectively) showed congestion and edema. The pulmonary section showed aspirated materials with bacterial packets, without an inflammatory reaction (Figure 4D).

**Figure 5:**
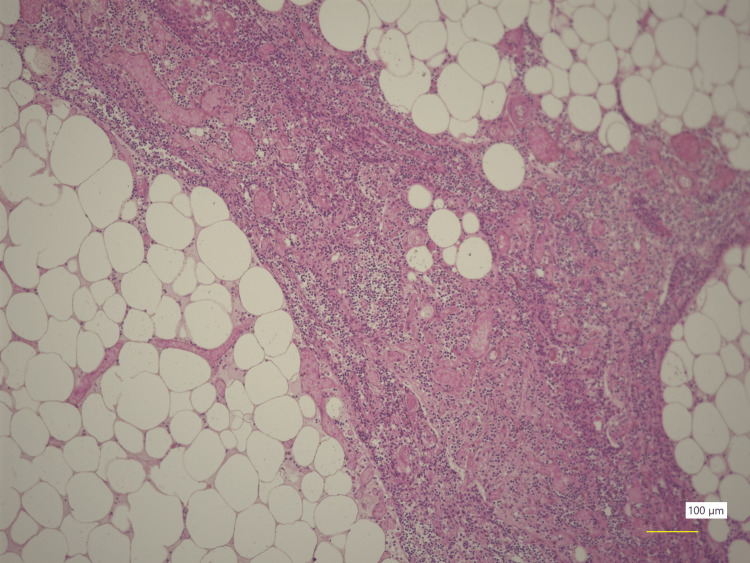
Thymus The thymus is generally fatty, but partially preserved.

The cardiac cavity contained a large number of coagula, and the renal cortical pallor of the deceased implied shock. Supporting the large liver volume detected by CT (Figure 1D), the enlarged fatty liver weighed 2,755 g. The autopsy findings revealed that the soft mediastinal mass observed on CT (Figure 2 and Figure 3) was fatty tissue and that the thymus (weighing 65 g) had been largely replaced with fatty tissue (Figure 5).

## Discussion

Tracheal obstruction

Here, we describe a young male with obesity who died unexpectedly while in the supine position due to tracheal compression caused by a large mediastinal fat mass. Low CT absorption suggested the presence of air and a patent upper trachea, but the absence of air in the lower trachea indicated obstruction by mediastinal soft tissue. Autopsy confirmed that the mediastinal soft tissue was fat-rich and that adipose tissue had replaced a large thymus. The PMCT findings showed that the lower trachea had been chronically obstructed between the mediastinal fatty mass and thoracic vertebrae. Upon excision, the lower trachea remained flattened but slowly regained a tubular shape. Histology confirmed normal tracheal cartilage morphology (data not shown), indicating sustained tracheal compression.

Given that the oxygen saturation was normal at a recent clinic visit, the trachea was likely compressed while sleeping in the supine position in which the deceased was found. Sagittal CT images showed posterior deviation and obstruction of the upper airway (Figure 2), possibly through glossoptosis, and reduced tone of the pharyngeal opening muscles, consistent with OSA. Supporting this latter interpretation, intermittent obstruction of the upper airway is known to be induced during rapid eye movement sleep, particularly in persons with obesity and reduced pharyngeal muscle tone [11]. Collectively, tracheal compression and OSA would have caused chronic hypoxia and intermittent hypoxia, respectively, and resulted in death while sleeping.

The lower esophagus was expanded with air (Figure 1), which is an established feature of achalasia caused by ganglion cell degeneration in the myenteric plexus of the esophageal body and lower esophageal sphincter [8]. However, histological findings ruled out achalasia as the cause of lower esophageal distention in this case. We concluded that the upper esophagus was compressed by the mediastinal fat tissue and acted as a check valve that allowed retention of swallowed air in the lower esophagus. This constricted the distal esophagus via leftward traction and compression by the enlarged fatty liver. The large fatty liver pushed the diaphragm upward, reducing the thoracic volume.

Overall, PMCT findings before autopsy and the finding of tracheal patency at autopsy helped ascertain the cause of death.

Cause of death

The PMCT and autopsy findings supported nocturnal chronic hypoxia due to tracheal compression and intermittent hypoxia resulting from OSA-induced sympathetic nerve activation [5,11,12]. Obesity and diabetes also promote sympathetic nerve activation [6,7]. Epidemiologically, obesity frequently causes type II diabetes and OSA, increasing the risk of hypertension, heart failure, coronary heart disease, atrial fibrillation, and ventricular tachycardia associated with QT prolongation [7,11,12]. The anatomical abnormalities resulting in the tracheal obstruction were postulated as the cause of the sudden death; however, the implications of morbid obesity, type II diabetes, and/or coronary arteriolar sclerosis in his death cannot be excluded. In addition, we have estimated the mechanism of sudden death, based on anatomical findings that were not directly corroborated with clinical findings like OSA, as well as respiratory and cardiac functions before death.

Sympathetic nerve activation accompanied by a catecholamine surge induces unexpected nocturnal death initially by increasing blood volume, stroke volume, cardiac motility, heart rate, and left ventricular and atrial volumes, and consequently inducing cardiac overload [13]. Second, catecholamine overflow promotes both cellular uptake of K+ via β2-adrenoceptor-mediated Na+, K+-ATPase activation and renal excretion of K+ via aldosterone over-secretion, which induces hypokalemia [13]. Third, catecholamine induces β-adrenoceptor-mediated myocardial Ca2+ overload [13]. Together with hypokalemia, the cardiac and myocardial Ca2+ overload induce heart failure and arrhythmias.

The intra-cardiac coagula and renal cortical pallor (shocked kidney) observed in this case suggest subacute, rather than sudden death. Diffuse pulmonary congestion and edema indicate heart failure, whereas diffuse myocardial fragmentation and waving reflect hypercontraction and hyperrelaxation, respectively [9]. Along with mild ventricular dilation, these findings suggest acute death from cardiogenic shock. The main coronary arteries were not sclerotic, but the walls of small intra-myocardial arteries were thickened (Figure 4), which is consistent with micro-circulatory dysfunction associated with diabetes. Histological findings revealed no myocardial infarction or cardiomyopathy. The trachea contained aspirate and lung histology showed a small amount of foreign material and a bacterial bolus. Given the absence of an accompanying inflammatory reaction around the foreign materials, we estimated that the aspiration occurred shortly before death and was not the primary cause of death.

Scene investigation showed that the deceased had pasted coolants on his skin, consumed several bottles of electrolytes, and set an air conditioner to 20℃ at a time when most residents in the vicinity would not have felt the need to use an air conditioner. These circumstances suggest excessive sweating due to feeling hot, although his body temperature at a clinic had been normal shortly before the estimated day of death. Hypertension was noted upon presentation at the clinic, and the detection of myocardial fragmentation histologically supports sympathetic activation. We hypothesized that excessive heat was produced from skeletal muscle hypermetabolism due to sympathetic nerve activation [14].

## Conclusions

The findings from PMCT and autopsy determined that the sudden death of a young male with obesity was due to tracheal compression while sleeping in the supine position caused by a mediastinal fat mass. His thoracic cavity was narrowed by a large fatty liver and an upper airway obstruction, possibly related to OSA, which contributed to hypoxia. These findings support hypoxia and are known to induce nerve activation.

Diabetes and OSA are prevalent in people who are morbidly obese and precede hypertension, heart failure, arrhythmias, and sudden cardiac death via sympathetic activation. In this case, sympathetic activation was supported by hypertension, with findings indicating exaggerated heat production and myocardial hyper-contraction. PMCT imaging in three planes before autopsy was useful for examining tracheal patency and estimating the cause and pathophysiology of death.
